# Genome Sequence of *Rhodococcus opacus* Strain R7, a Biodegrader of Mono- and Polycyclic Aromatic Hydrocarbons

**DOI:** 10.1128/genomeA.00827-14

**Published:** 2014-08-21

**Authors:** P. Di Gennaro, J. Zampolli, I. Presti, M. Cappelletti, P. D’Ursi, A. Orro, A. Mezzelani, L. Milanesi

**Affiliations:** a Department of Biotechnology and Biosciences, University of Milano-Bicocca, Milan, Italy; b Department of Pharmacy and Biotechnology (FaBiT), University of Bologna, Bologna, Italy; c CNR, Institute for Biomedical Technologies, Segrate, Italy

## Abstract

*Rhodococcus opacus* strain R7 (CIP107348) degrades several mono- and polycyclic aromatic hydrocarbons. Here, we present the high-quality draft genome sequence of strain R7, consisting of 10,118,052 bp, with a G+C content of 67.0%, 9,602 protein-coding genes, and 62 RNAs genes.

## GENOME ANNOUNCEMENT

Members of the genus *Rhodococcus* demonstrate a remarkable ability to degrade a wide range of natural organic and xenobiotic compounds (1). *Rhodococcus opacus* R7 (CIP107348) was isolated from a polycyclic aromatic hydrocarbon-contaminated site in Italy because of its ability to grow on naphthalene and *o*-xylene as the only carbon and energy source (2, 3). As R7 also catabolizes a wide range of aliphatic, alicyclic, and both mono- and polycyclic aromatic hydrocarbons, it represents a strain of considerable environmental and industrial interest.

The genome sequencing of *Rhodococcus opacus* R7 was performed using 454 sequencing technology (Roche GS FLX Titanium). The total numbers of sequence reads were 312,384 from one shotgun library and 380,920 from one paired-end library. All the reads were assembled using Newbler 2.6 into 223 contigs, with an *N*_50_ length of 184,729 bp and an average genome coverage of 17×. Based on paired-end directional information, the contigs were further ordered into 6 scaffolds, giving a total genome size of 10.1 Mb with a G+C content of 66.0%.

The annotation was performed by using the RAST (Rapid Annotation using Subsystem Technology) server (4). Five scaffolds, constituting a total of ~1.65 Mb, are likely to be plasmids, as they carry genetic signatures typical of *Rhodococcus* plasmids.

A total of 9,602 putative open reading frames (ORFs), 62 RNAs genes, 9 rRNAs and 53 tRNAs, were predicted. RAST annotation indicates that strains *Rhodococcus jostii* RHA1 (score 501), *Rhodococcus opacus* B4 (score 409) are the closest neighbors of strain R7. Subsystem (457) categories representing the metabolisms of carbohydrates, amino acids and cofactors, vitamins, prosthetic groups, or pigments are the most abundant, and they account for 1,236, 995, or 665 proteins, respectively. A total of 745 ORFs are involved in metabolism of fatty acids, lipids, and isoprenoids, while 267 ORFs participate in metabolism of aromatic compounds. A total of 129 oxygenases/hydroxylases among the 134 annotated are predicted to initiate oxidation of organic compounds with industrial and environmental relevance, such as linear alkanes, cyclic ketones, aromatic compounds (e.g., benzoate, catechol, gentisate, salycilate, and byphenil), aminopolycarboxilic acids, nitroalkanes, and phenylalkanoic acids. Forty-five ORFs encode cytochrome P450 monooxygenases that catalyze regio- and stereospecific oxidation of a vast number of substrates (5).

### Nucleotide sequence accession numbers.

This whole-genome shotgun project has been deposited at DDBJ/EMBL/GenBank under the accession numbers CP008947, CP008948, CP008949, CP008950, CP008951, and CP008952. The versions described in this paper are the first versions.
